# Supplementary material for: Identification of photoperiod-induced specific miRNAs in the adrenal glands of Sunite sheep (Ovis aries)
Source: Front Vet Sci. 2022 Jul 22;9:888207. doi: 10.3389/fvets.2022.888207 (PMC9354845; doi:10.3389/fvets.2022.888207)
Supplement: Supplementary file 1 [file Data_Sheet_1.ZIP › Supplementary file/miRDeep2 file.zip!/miRDeep2 file/Know_Structure/LP42A3/oar-mir-380.pdf]

miRBase precursor : oar-mir-380
Total read count : 4373
oar-miR-380-5p read count : 70
oar-miR-380-3p read count : 4302
remaining reads : 1

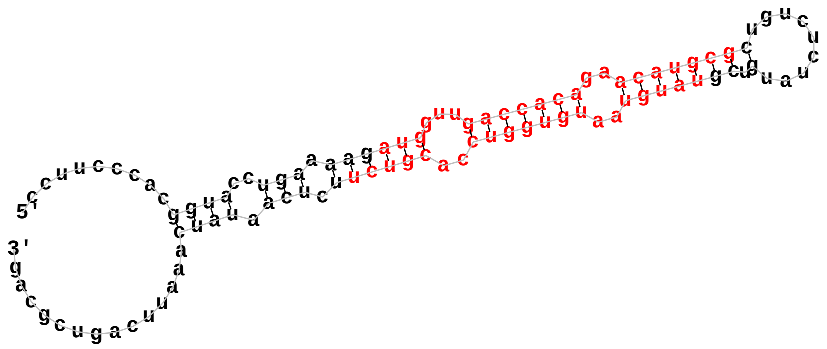

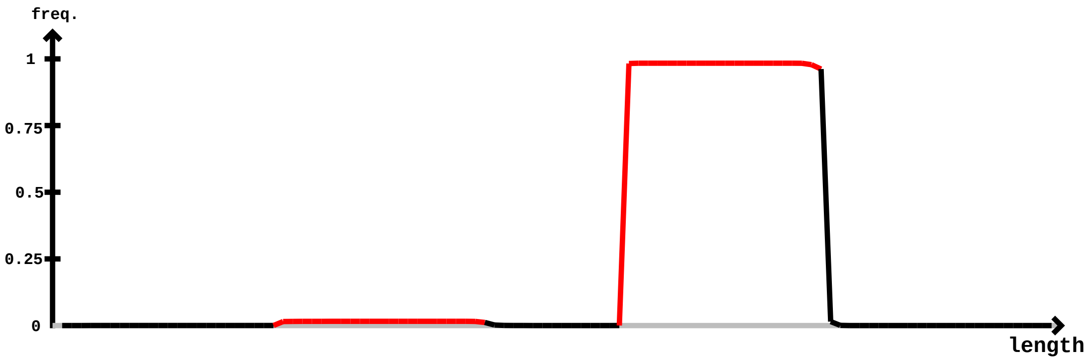

oar-miR-380-5p

oar-miR-380-3p

5'- ccuucccacgguaccugaaaagaugguugaccacagaacaugcgcugucucuaugucguauguaaugugguccacgucuucucaauaucaaauucagucgcag -3'	exp		
........((((..(((.((((((...(((((((..((((((((.........).))))))).)))))))..)))))).))).))))..............	reads	mm	sample
..uucccacgguaccugaaaag...	1	0	seq
......................augguugaccacagaacaug...	2	0	seq
......................augguugaccacagaacaugc..	16	0	seq
......................augguugaccacagaacaugcg...	41	0	seq
......................augguugaccacagaacaugcgc..	7	0	seq
.......................ugguugaccacagaacaugc..	1	0	seq
.......................ugguugaccacagaacaugcg...	1	0	seq
........................gguugaccacagaacaugcgcu...	1	0	seq
...........................ugaccacagaacaugcgcugu...	1	0	seq
....guauguaaugugguccacgucu........................	1	0	seq
....uauguaaugugguccacg...........................	1	0	seq
....uauguaaugugguccacgu..........................	23	0	seq
....uauguaaugugguccacguc.........................	72	0	seq
....uauguaaugugguccacgucu........................	4135	0	seq
....uauguaaugugguccacgucuu.......................	62	0	seq
....uauguaaugugguccacgucuuc......................	3	0	seq
....auguaaugugguccacgucu........................	5	0	seq
